# Prescription of secondary preventive drugs after ischemic stroke: results from the Malaysian National Stroke Registry

**DOI:** 10.1186/s12883-017-0984-1

**Published:** 2017-11-23

**Authors:** Wen Yea Hwong, Zariah Abdul Aziz, Norsima Nazifah Sidek, Michiel L. Bots, Sharmini Selvarajah, L. Jaap Kappelle, Sheamini Sivasampu, Ilonca Vaartjes

**Affiliations:** 1 National Clinical Research Centre, Ministry of Health Malaysia, Kuala Lumpur, Malaysia; 2 Julius Center for Health Sciences and Primary Care, University Medical Center Utrecht, Utrecht, The Netherlands; 3 Department of Neurology, Hospital Sultanah Nur Zahirah, Kuala Terengganu, Terengganu Malaysia; 4 Clinical Research Centre, Hospital Sultanah Nur Zahirah, Kuala Terengganu, Terengganu Malaysia; 5 Sharmini Selvarajah Consulting, Selangor, Malaysia; 6 Department of Neurology and Neurosurgery, Brain Center Rudolf Magnus, University Medical Center Utrecht, Utrecht, The Netherlands

**Keywords:** Brain ischemia, Secondary prevention, Platelet aggregation inhibitors, Anticoagulants, Hydroxymethylglutaryl-CoA reductase inhibitors, Antihypertensive agents

## Abstract

**Background:**

Evaluation of secondary stroke prevention in low and middle-income countries remains limited. This study assessed the prescription of secondary preventive drugs among ischemic stroke patients upon hospital discharge in Malaysia and identified factors related to the prescribing decisions.

**Methods:**

From Malaysian National Stroke Registry, we included patients with non-fatal ischemic stroke. Prescriptions of antiplatelet, anticoagulants, antihypertensive drugs and lipid-lowering drugs were assessed. Multi-level logistic regressions were performed to determine the relation between potential factors and drug prescriptions.

**Results:**

Of 5292 patients, 48% received antihypertensive drugs, 88.9% antiplatelet and 88.7% lipid-lowering drugs upon discharge. Thirty-three percent of patients with an indication for anticoagulants (*n* = 391) received it. Compared to patients <=50 years, patients above 70 years were less likely to receive antiplatelet (OR: 0.72, 95% CI: 0.50–1.03), lipid-lowering drugs (OR: 0.66, 95% CI: 0.45–0.95) and anticoagulants (OR: 0.27, 95% CI: 0.09–0.83). Patients with moderate to severe disability upon discharge had less odds of receiving secondary preventive drugs; an odds ratio of 0.57 (95% CI: 0.45–0.71) for antiplatelet, 0.86 (95% CI: 0.75–0.98) for antihypertensive drugs and 0.78 (95% CI: 0.63–0.97) for lipid-lowering drugs in comparison to those with minor disability. Having prior specific comorbidities and drug prescriptions significantly increased the odds of receiving these drugs. No differences were found between sexes and ethnicities.

**Conclusions:**

Prescription of antihypertensive drugs and anticoagulants among ischemic stroke patients in Malaysia were suboptimal. Efforts to initiate regular clinical audits to evaluate the uptake and effectiveness of secondary preventive strategies are timely in low and middle-income settings.

**Electronic supplementary material:**

The online version of this article (10.1186/s12883-017-0984-1) contains supplementary material, which is available to authorized users.

## Background

There is a substantial geographical variation for stroke burden between countries of different income levels. The bulk of stroke burden comes from low and middle-income countries (LMIC), accounting for 69% of total incident strokes and 71% of stroke deaths in 2010. The number of disability-adjusted life years (DALYs) for stroke survivors aged below 75 years was 5-times higher in these regions compared to high-income countries [1].

The burden of stroke in LMIC is largely attributable to poor prevention and control of cardiovascular risk factors [2]. Besides primary prevention, part of the burden is potentially modifiable with effective secondary prevention. Stroke patients have a 30% estimated 5-year risk of a recurrent stroke [3]. Regular clinical audits are established to assess control of cardiovascular risk factors for secondary prevention [4] but such structures are particularly lacking in LMIC. Nevertheless, this information is essential because the implementation and uptake of secondary prevention in LMIC is likely to differ from high-income countries, owing to the disparity in terms of access to health care, average education level and availability of drugs between countries of different income statuses [5].

This study was therefore aimed at evaluating the prescription of secondary preventive drugs upon hospital discharge among ischemic stroke patients in Malaysia, an upper middle-income country. Furthermore, we sought to identify possible factors that influence the likelihood of patients being prescribed with these drugs.

## Methods

### Participant selection

Participants for this study were selected from cases that were registered in Malaysian National Stroke Registry, a database established under the National Neurology Registry [6]. This database recorded a total of 7592 patients from 14 public hospitals between July 2009 and December 2014. Although the coverage of this registry does not include stroke admissions from private hospitals, this database is the best available representation of the Malaysian stroke population. Moreover, public hospitals cover 66.2% of total hospital admissions for the country in 2014 [7].

For the present study, we included patients who had a diagnosis of non-fatal ischemic stroke upon discharge.

### Secondary stroke prevention

We kept to recommendations of the 2012 Malaysian Clinical Practice Guidelines for Management of Ischemic Stroke [8]. The contents are largely similar to the 2011 stroke guidelines published by American Stroke Association [9]. We did not account for evidence from an updated publication for the latter guidelines in 2014. This is to establish a consistency in terms of time frame between dissemination of information from the guidelines and selection of patients for this study.

Prescriptions of three types of drugs were assessed: 1) antithrombotic drugs comprising antiplatelet and anticoagulants; 2) antihypertensive drugs; and 3) lipid-lowering drugs. Indications for long-term anticoagulants in the present analysis included prior history of atrial fibrillation, electrocardiogram showing atrial fibrillation during admission or patients with cardioembolic stroke. Drugs were prescribed upon hospital discharge by treating physicians. Data for the prescription were obtained from medical records. These drugs were coded following the Anatomical Therapeutic Chemical (ATC) Classification, an international classification under the World Health Organization of which drugs are coded based on their active ingredients [10].

### Determinant measures

Potential patient-level factors that were studied included 1) demographic characteristics: age, sex, ethnicity and education level; 2) comorbidities and prescriptions recorded prior to the stroke event: previous hypertension, diabetes mellitus, dyslipidemia, atrial fibrillation, ischemic heart disease, previous stroke or transient ischemic attack (TIA) events and prior prescriptions of antiplatelet, anticoagulants, antihypertensive drugs and lipid-lowering drugs; 3) lifestyle factors: obesity and smoking status and 4) status of disability upon discharge that was assessed with Modified Rankin Scale (mRS). A hospital level variable that categorizes hospitals into state and non-state hospitals was also included. State hospitals are classified as hospitals with up to 45 resident specialties or subspecialties and are normally main referral centers for each state. Detailed operationalization of these factors are summarized in Additional file 1: Table S1.

Collection of data for this registry followed local routine clinical practice where it involved collection of existing data that were readily available as part of routine practice. Information on demographic characteristics with the exception of education level was obtained from patient identification cards. Education level was assessed via patient interview. Depending on availability, information on prior co-morbidities, lifestyle factors and drug prescriptions obtained during patient interviews were verified with patients’ past medical records from respective general practitioners or availability of drug strips from patients. Clinical diagnosis of an ischemic stroke was confirmed via computed tomographic (CT) imaging where a visible infarct seen on scan confirmed an ischemic stroke. As for disability status, it was measured prior to discharge with mRS. This is a commonly used scale that incorporates both mental and physical adaptations to neurological deficits experienced after a stroke [11, 12]. For the purpose of this study, a score of <3 was considered no or minor disability and a score of > = 3 as moderate or severe disability.

### Statistical analysis

Proportion of missing data ranged from 0.06% (variable: ischemic heart diseases) to 39% (variable: smoking status). Details on the extent of missingness for each variable are summarized in Additional file 1: Table S1. Missing data were assumed to be missing-at-random and thus, we conducted multiple imputation with m = 10 prior to the inclusion of patients for analysis to reduce the extent of bias resulting from missing data [13]. Subsequently, patients who did not fulfil the inclusion criteria were removed from analysis.

As stroke patients were selected within clusters of hospitals, the variable ‘Hospital ID’ that defines each participating hospital was included as a random effect, to account for variations within and between hospitals. In that respect, multi-level logistic regressions were performed to identify factors that were associated with the likelihood of an ischemic stroke patient being prescribed antiplatelet, antihypertensive drugs and lipid-lowering drugs, respectively. All potential factors were fitted into regression models for the three drugs. Factors with *p*-value <0.05 were considered significant. For anticoagulants, a slightly different model fitting approach was taken as the number of prescriptions (events) were small (*n* = 128). Univariable analysis for each factor was first conducted to assess its possible relation to the outcome. Factors with *p*-value < 0.25 were chosen and subsequently included in a multivariable analysis.

Multiple imputation was conducted with package ‘mice’ in R version 3.1.1 [14]. Multi-level regression analyses were performed with Stata SE Version 14.3 [15]. Odds ratios with their corresponding 95% confidence intervals were reported.

## Results

### Patient characteristics

Of 5292 patients, 43% were 60 years old or younger. A majority were of Malay ethnicity and nearly half of the patients had primary education. Table 1 shows that presence of atherosclerotic risk factors was common; 72% had prior hypertension, 47% diabetes mellitus and 32% dyslipidemia. Only 7% had atrial fibrillation. Prior to their hospital admission for ischemic stroke, 40% of patients were receiving antihypertensive drugs, 24% antiplatelet, 28% lipid-lowering drugs and 2%, anticoagulants. Among those with hypertension, 52% (*n* = 1960) were taking antihypertensive drugs. Upon hospital discharge, 62% had moderate to severe disability.

**Table 1:** Patient characteristics

Characteristics	Patients Included (*N* = 5292)
	n (%)
Age category (years)	
< =50	855 (16)
51–60	1413 (27)
61–70	1544 (29)
> 70	1480 (28)
Sex	
Men	2913 (55)
Women	2379 (45)
Ethnic group	
Malay	4392 (83)
Non-Malay	900 (17)
Education level	
Nil	936 (18)
Primary	2455 (46)
Secondary	1685 (32)
Tertiary	216 (4)
Co-morbidities prior to admission	
Hypertension	3798 (72)
Diabetes mellitus	2462 (47)
Dyslipidemia	1665 (32)
Atrial fibrillation^a^	346 (7)
Ischemic heart disease	686 (13)
Previous stroke/TIA^b^ events	1166 (22)
Life-style factors	
Smoking	
Never smoked	2634 (50)
Previous smoker (quit >30 days)	1024 (19)
Current smoker	1634 (31)
Obesity	343 (7)
Drug use prior to admission	
Antiplatelet	1283 (24)
Anticoagulants	96 (2)
Antihypertensive drugs	2117 (40)
Lipid-lowering drugs	1490 (28)
Disability scale at discharge (mRS^b^)	
< 3	2028 (38)
> =3	3264 (62)

^a^includes patients with documented history of atrial fibrillation and patients with electrocardiogram showing atrial fibrillation during admission for ischemic stroke

^b^TIA = transient ischemic attack; mRS = Modified Rankin Scale

### Prescription of secondary preventive drugs

**Fig. 1:**
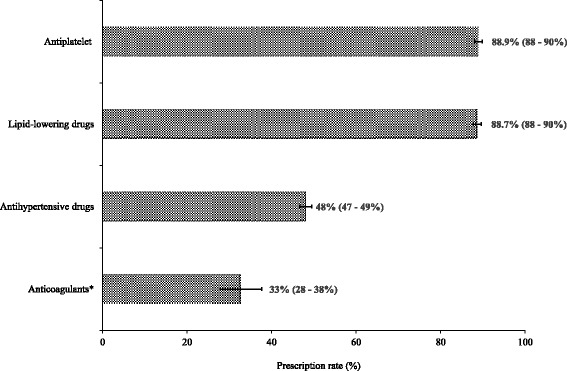
Prescription of secondary preventive drugs. *for ischemic stroke patients with indications to receive anticoagulants (*n* = 391)

Figure 1 shows that 88.9% (95% CI: 88–90%) were discharged with antiplatelet. Similarly, 88.7% (95% CI: 88–90%) of patients received lipid-lowering drugs. Forty-eight percent (95% CI: 47–49%, *n* = 2543) of patients were discharged with antihypertensive drugs. Of those, 62% of them were on monotherapy. Angiotensin-converting-enzymes inhibitors (ACEIs) recorded the highest number of prescriptions (65%) whereas ACEIs and calcium channel blockers were the most commonly prescribed dual combination.

**Fig. 2:**
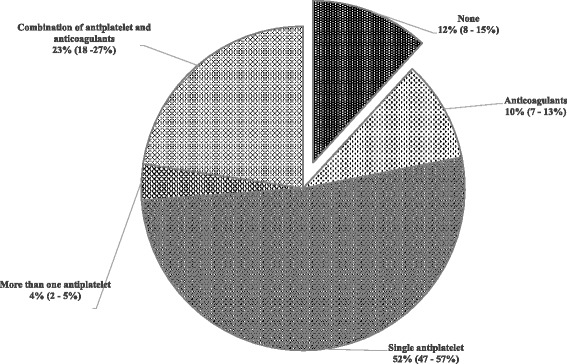
Types of antithrombotic drugs prescribed among ischemic stroke patients with indications for anticoagulation. (*n* = 391). *exploded slice from the pie chart indicates the proportion of patients who were not prescribed with any antithrombotic drugs

Among ischemic stroke patients with an indication for long-term anticoagulants (*n* = 391), 33% (95% CI: 28–38%) received it. Figure 2 illustrates the types of antithrombotic drugs prescribed. More than half (52%, 95% CI: 47–57%) were discharged with a single antiplatelet and 4% (95% CI: 2–5%), more than one antiplatelet. There were 12% (95% CI: 8–15%) of the indicated patients who did not receive any antithrombotic drugs.

### Factors associated with the prescription of secondary preventive drugs (Fig. 3a–d and Additional file 2: Table S2)

**Fig. 3:**
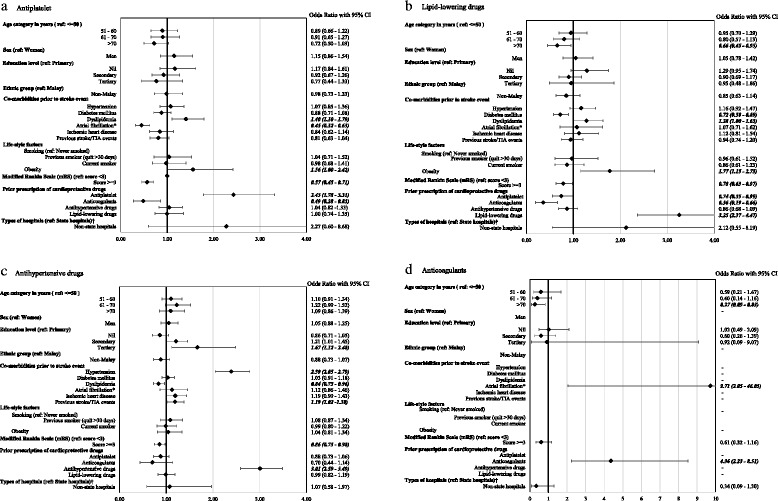
Factors related to the prescription of secondary preventive drugs among ischemic stroke patients. (Fig. 3 **a**–**d** are results from multivariable analyses, ref. = reference groups, TIA = transient ischemic attack, mRS = Modified Rankin Scale, − = factors were not included in multivariable analysis). *atrial fibrillation includes patients with documented history of atrial fibrillation and patients with electrocardiogram showing atrial fibrillation during admission for ischemic stroke. †state hospitals refer to hospitals with up to 45 resident specialties or subspecialties and are normally main referral centers for each state

Increasing age was associated with less odds of receiving secondary preventive drugs (Fig. 3a–d). Patients above 70 years old were less likely to be discharged with secondary preventive drugs when compared to those aged 50 years and below; an odds ratio of 0.72 (95% CI: 0.50–1.03) for antiplatelet, 0.66 (95% CI: 0.45–0.95) for lipid-lowering drugs and 0.27 (95% CI:0.09–0.83) for anticoagulants. Contrastingly, this association was not observed for the prescription of antihypertensive drugs.

Figure 3a–d displays no differences in the odds of receiving secondary preventive drugs between men and women or their ethnicities. In contrast, patients with moderate to severe disability (mRS > =3) were less likely to be discharged with antiplatelet (OR: 0.57, 95% CI: 0.45–0.71), antihypertensive drugs (OR: 0.86, 95% CI: 0.75–0.98) and lipid-lowering drugs (OR: 0.78, 95% CI: 0.63–0.97) in comparison to those with minor disability (mRS < 3). This observation was also noted for the prescription of anticoagulants although the association was not significant (OR: 0.61, 95% CI: 0.32–1.16).

Some factors were found to be associated with specific to respective drug prescriptions. Patients with secondary and tertiary education were more likely to be prescribed antihypertensive drugs when compared to those who completed primary education (OR: 1.21, 95% CI: 1.01–1.45 for the former and OR: 1.67, 95% CI: 1.12–2.48 for the latter). There was a 2.4-fold increase (95% CI: 2.05–2.79) in the prescription of antihypertensive drugs in patients with prior hypertension and similarly in those with previous TIA or ischemic stroke, a 19% increase (95% CI: 1.02–1.38) was noted (Fig. 3c).

Patients with dyslipidemia had significantly higher odds of receiving antiplatelet (OR: 1.40, 95% CI: 1.10–1.79) and lipid-lowering drugs (OR: 1.28, 95% CI: 1.00–1.63). Likewise, patients who were obese at admission were also more likely to be prescribed these drugs; an odds ratio of 1.56 (95% CI: 1.00–2.42) for antiplatelet and OR: 1.77 (95% CI: 1.15–2.73) for lipid-lowering drugs. Besides, our findings revealed a 9.7-fold increase (95% CI: 2.05–46.05) in the odds of being discharged with anticoagulants among ischemic stroke patients with atrial fibrillation. Figure 3a–d shows that the odds of receiving secondary preventive drugs significantly increased between 2 to 4-fold with prior respective drug prescription.

In addition, we found a 16% decrease (95% CI: 0.73–0.96) in the odds of receiving antihypertensive drugs in patients with dyslipidemia and similarly, a 28% decrease (95% CI: 0.58–0.89) in the odds of receiving lipid-lowering drugs in diabetic patients. Patients with prior antithrombotic drugs were also less likely to be prescribed with lipid-lowering drugs.

## Discussion

Prescription of antihypertensive drugs and anticoagulants upon hospital discharge among ischemic stroke patients in Malaysia were suboptimal. Less than half of the ischemic stroke patients were prescribed antihypertensive drugs and only 1 out of 3 ischemic stroke patients with an indication for long-term anticoagulants received the drug. Increasing age and poorer disability status consistently decreased the odds of receiving secondary preventive drugs upon hospital discharge whereas patients with specific comorbidities prior to admission and who were previously taking the respective drugs were more likely to be treated for secondary prevention. Importantly, prescription of these secondary preventive drugs was not influenced by different sexes and ethnicities.

The high proportion of patients receiving antiplatelet was consistent with previous studies [16–19]. In contrast, a majority of studies reported low prescriptions of lipid-lowering drugs with a range between 31 and 45% [16, 17, 19], with the exception of Thailand [18]. Inclusion of statin prescription as a key performance indicator for stroke management in the country and similarly, in Thailand may explain the larger proportions observed [8, 18].

The low rate of in-hospital initiation of antihypertensive drugs after an ischemic stroke event however, warrants crucial attention. Comparatively, the proportion of antihypertensive drugs received varied within LMIC regions; from 31% in Thailand [18] to 63% in China [19] whereas a higher range between 69 and 77% were observed in high-income countries [17, 20]. While fluctuations in the measurement of blood pressure during hospital admission may had possibly led to a delay in prescription among some patients, there were potentially other contributing factors to the fact that more than half of the patients in our cohort were discharged without an antihypertensive drug. Of importance is the uncertainty to prescribe these drugs to normotensive patients after an ischemic stroke and the extent of lowering their blood pressure levels [21]. Despite this contentious issue, the local guidelines, supported by the international guidelines on secondary stroke prevention in 2011 have recommended the use of antihypertensive drugs, in particular ACE inhibitors as part of secondary preventive therapy in both hypertensive and normotensive patients [8, 9]. Moreover, Thompson et al. [22] showed a significant reduction in the risk of recurrent stroke for non-hypertensive patients who were prescribed antihypertensive drugs (RR: 0.77; 95%CI: 0.61–0.98). It is essential to be aware that initiation of secondary stroke prevention goes beyond solely treating specific risk factors. In addition, despite an increase in the prevalence of hypertension in Malaysia, treatment, awareness and control of the condition remains alarmingly low [23]. This is clearly observed from our findings where only half of the hypertensive patients received antihypertensive drugs prior to admission.

Besides that, the influence of education level on the likelihood of being prescribed antihypertensive drugs upon hospital discharge is of particular interest. The decision to prescribe is often a complex interplay between the prescriber, patient and resources available. Used as a proxy for socioeconomic status and literacy level, patients of higher education levels are more likely to understand the benefits attained with secondary prevention and thus, showed more acceptance towards treatment [24].

Parallel to our findings, a range between 19 and 40% for the prescription of anticoagulants was reported among Asian cohorts in LMIC [18, 25]. Fear of intracranial bleeding as well as difficulties in achieving optimal anticoagulation with warfarin especially among elderly Asians might play a role in the suboptimal prescription of long-term anticoagulants [26]. This is reflected in our findings where younger patients with lower risk of bleeding and those who received the drugs before, were more likely to receive anticoagulants. Locally, the current delivery of stroke care is fragmented, in particular between the points of transfer of care. Besides a lack of post-discharge stroke care guidelines in primary care, resources are restricted especially in rural areas. Follow-up services for post-stroke patients therefore, largely remain within the settings of tertiary centres that are located in the main cities [27]. Nonetheless, this poses several challenges in terms of distance, logistics and convenience for patients. Initiation of treatment such as warfarin that requires frequent monitoring of prothrombin time and dose readjustments are often not possible.

Increase in age and higher mRS score are predictors of recurrent vascular events [28]. While rightfully patients with higher risks of stroke recurrence should be more optimally treated, we found that older patients and those with worse disability were less likely to receive secondary preventive drugs. Although previous studies reported conflicting results on prescription of secondary preventive drugs in older patients, under prescription in older patients could be attributed to issues on increased adverse effects [16]. Comparatively, findings on the relation between disability status and drug prescription were similar to other studies [19, 25]. Restrictions in drug availability within some LMIC may explain such practice but more importantly, this is a reflection of the difficulties faced by physicians in providing the best ‘do no harm’ care to patients. Patients with poor independence status are generally those with a higher number of comorbidities. Pill burden may cause non-adherence and an increase risk of adverse drug reactions [29]. Moreover, secondary prevention is perhaps viewed as of little value for these patients because they are perceived to have less to lose with future recurrent events.

Furthermore, we found no differences in the odds of receiving secondary preventive drugs between sexes and ethnicities of the patients. In developing countries where issues of gender and ethnic discriminations ranging from job choices, societal expectations to limited access to education and healthcare are often raised [30], absence of such associations are reassuring facts that these social determinants do not influence a prescriber’s decision to treat.

Exact reasons remain to be ascertained so as to why there were less odds in prescribing antihypertensive drugs among patients with prior dyslipidemia and similarly, lipid-lowering drugs for diabetic patients and patients with prior antithrombotic drugs. We postulate the probability of variations in blood pressure and lipid levels after an acute ischemic stroke that can potentially delay the prescription of antihypertensive drugs [31]. Statin precautions such as fear of increased risk for intracranial bleed or raised liver enzymes in diabetic patients with fatty liver disease are among possible reasons [32, 33] Nevertheless, recent evidences are increasingly proving the greater benefits of these drugs over their minimal risks [34]. Thus, unless for absolute contraindications, there is no reason to withhold secondary preventive drugs from these indicated patients.

To the best of our knowledge, we are among the few LMIC regions within Southeast Asia to initiate evaluation of secondary stroke prevention. Our large study sample is of advantage. In addition, we performed multiple imputations to reduce possible bias from missing data. It was not possible, however to identify patients with absolute contraindications to the drugs because reasons for non-prescription were unknown. Comorbidities upon hospital discharge or its proxy measurements were unavailable. Besides, this study is not designed to be nationally representative. Caution should be taken when generalizing the findings to the whole Malaysian stroke population.

Fundamentally, this study sets a benchmark of the current status of secondary stroke care among ischemic stroke patients in Malaysia. These findings imply that it is timely to establish regular assessment on the uptake and effectiveness of these preventive strategies. Establishing collaborations with other countries will allow initiation of such evaluation to be carried out. Among the few examples are the conduct of EUROASPIRE and Survey of Risk Factors audit (SURF) where a standardized method of assessing secondary prevention for cardiovascular diseases is implemented across participating countries to enable comparisons and benchmarking [4, 35]. Findings from the assessment should subsequently be put into use by initiating a continuous feedback system to relevant healthcare professionals and stakeholders. Besides, the need for an improved stroke care model in the country with an emphasis on continuity of care for post-stroke patients at various levels of care should be made a priority. Other targeted plans include organizing regular continuous medical updates to increase prescribers’ awareness on the impact of in-hospital initiation of secondary preventive drugs on subsequent improvement in patient adherence and overall survival [36].

## Conclusions

In summary, the prescription of antihypertensive drugs and anticoagulants upon hospital discharge among ischemic stroke patients in Malaysia were suboptimal. Our findings revealed a treatment pattern that was influenced by age, disability upon discharge, specific comorbidities and prior drug prescriptions. No differences were found between sexes and ethnicities. Continuous efforts from relevant aspects of healthcare stakeholders are essential to allow better cardiovascular preventive actions to be put into place.

## Additional files

Additional file 1: Table S1. Operationalization and proportion of missingness for determinants. This table shows definitions for each variable included in the regression analysis, the types of variables and its proportion of missingness in the dataset. (DOCX 19 kb)

Additional file 2: Table S2. Factors related to the prescription of secondary preventive drugs among ischemic stroke patients by types of drugs (multivariable analysis). This table is similar to Fig. 3a–d but the estimates are provided in a table format. (DOCX 21 kb)

## Abbreviations

ATC: Anatomical Therapeutic Chemical
CT: Computed tomographic
DALYs: Disability-adjusted-life-years
LMIC: Low and middle-income countries
mRS: Modified Rankin Scale
TIA: Transient ischemic attack
