# Supplementary material for: Prescription of secondary preventive drugs after ischemic stroke: results from the Malaysian National Stroke Registry
Source: BMC Neurol. 2017 Nov 23;17:203. doi: 10.1186/s12883-017-0984-1 (PMC5701494; doi:10.1186/s12883-017-0984-1)
Supplement: Supplementary file 1 — Operationalization and proportion of missingness for determinants. This table shows definitions for each variable included in the regression analysis, the types of variables and its proportion of missingness in the dataset. (DOCX 19 kb) [file 12883_2017_984_MOESM1_ESM.docx]

Additional Table 1. Operationalization and proportions of missingness for determinants

| Characteristics | Type of variable | Proportion of missing data (%)* | Operationalization |
| --- | --- | --- | --- |
| Age | Nominal | 0.4 | measured in years from the date of birth to the date of admission. |
|  |  |  |  |
|  |  |  | categorized to 4 groups: <=50, 51 - 60, 61 - 70 and >70 |
| Sex | Nominal | 0 | dichotomous variable: men and women |
| Ethnic group | Nominal | 0 | dichotomous variable: Malay and non-Malay |
| Education level | Nominal | 18 | measured by the highest education attained: none, primary education refers to six years of elementary education, secondary education refers to five years of high school education and tertiary education refers to completed education in institutions of higher learning |
| Co-morbidities |  |  |  |
| Hypertension | Nominal | 0 | documented history of hypertension, use of antihypertensive medications or recorded blood pressure of >140/>90 on at least two occasions prior to stroke event: yes and no |
| Diabetes Mellitus | Nominal | 0 | documented history of confirmed diagnosis of diabetes mellitus type 2 prior to stroke event: yes and no |
| Dyslipidemia | Nominal | 0 | documented history of confirmed dyslipidemia via elevated lipid levels prior to stroke event: yes and no |
| Atrial Fibrillation | Nominal | 0 | documented history of atrial fibrillation prior to or during stroke event: yes and no |
| Ischemic heart diseases | Nominal | 0.06 | documented history of ischemic heart disease prior to stroke event: yes and no |
| Previous stroke/TIA events | Nominal | 0.8 | documented history of previous stroke or TIA episodes: yes and no |
| Life-style factors |  |  |  |
| Smoking status | Nominal | 39 | categorized into 3 categories of smoking: 1 refers to never smoked before prior to the stroke event; 2 refers to former smoking history (quitted more than 30 days); and 3 refers to current smoking |
| Obesity | Nominal |  | measured by body mass index of >25kg/m2 (for Asian population)^1^: yes and no |
| Disability scale, Modified Rankin Scale (mRS) | Nominal | 0.3 | polychotomous scale which ranges from 0 (no symptoms) to 5 (severe disability). Dichotomized at a cut-off of 3; <3 and >=3 for this study |
| Prescription of cardioprotective drugs prior to admission |  |  |  |
| Antiplatelet | Nominal | 0.1 | Prescribed medication prior to the stroke event coded with ATC code B01AC: yes and no |
| Lipid lowering drugs | Nominal | 0.3 | Prescribed medication prior to the stroke event coded with ATC code C10: yes and no |
| Antihypertensive drugs | Nominal | 0.1 | Prescribed medication prior to the stroke event with ATC code of either C09, C08, C07, C03, C02: yes and no |
| Anticoagulants | Nominal | 0.1 | Prescribed medication prior to the stroke event coded with ATC code B01A except B01AC: yes and no |
| Types of hospital | Nominal | 0 | Dichotomous variable categorizing hospitals into a state hospital or not: yes and no (state hospitals are classified as hospitals with up to 45 resident specialties or subspecialties and are normally main referral centers for each state) |

^1^ International Obesity Taskforce. The Asia-Pacific perspective: Redefining Obesity and its treatment. World Health Organization Western Pacific Region. February 2000

*proportion derived from dividing count of missingness for each variable with the total number of patients in the analysis (N=5292 patients)
