# Supplementary material for: Prescription of secondary preventive drugs after ischemic stroke: results from the Malaysian National Stroke Registry
Source: BMC Neurol. 2017 Nov 23;17:203. doi: 10.1186/s12883-017-0984-1 (PMC5701494; doi:10.1186/s12883-017-0984-1)
Supplement: Supplementary file 2 — Factors related to the prescription of secondary preventive drugs among ischemic stroke patients by types of drugs (multivariable analysis). This table is similar to Fig. 3a–d but the estimates are provided in a table format. (DOCX 21 kb) [file 12883_2017_984_MOESM2_ESM.docx]

Additional Table 2. Factors related to prescription of secondary preventive drugs among ischemic stroke patients by types of drugs

|  | **Odds ratio with 95% CI** | | | |
| --- | --- | --- | --- | --- |
| **Factors** | **Antiplatelet** | **Lipid-lowering drugs** | **Antihypertensive drugs** | **Anticoagulants** |
| Age category (years) |  |  |  |  |
| <=50 | 1.00 (-) | 1.00 (-) | 1.00 (-) | 1.00 (-) |
| 51 - 60 | 0.89 (0.66 - 1.22) | 0.95 (0.70 - 1.29) | 1.10 (0.91 - 1.34) | 0.59 (0.21 - 1.67) |
| 61 - 70 | 0.91 (0.65 - 1.27) | 0.80 (0.57 - 1.13) | 1.22 (0.99 - 1.52) | 0.40 (0.14 - 1.16) |
| >70 | 0.72 (0.50 - 1.03) | ***0.66 (0.45 - 0.95)*** | 1.09 (0.86 - 1.39) | ***0.27 (0.09 - 0.83)*** |
| Sex |  |  |  |  |
| Women | 1.00 (-) | 1.00 (-) | 1.00 (-) | - |
| Men | 1.15 (0.86 - 1.54) | 1.05 (0.78 - 1.42) | 1.05 (0.88 - 1.25) |  |
| Education level |  |  |  |  |
| Nil | 1.17 (0.84 - 1.61) | 1.29 (0.95 - 1.74) | 0.86 (0.71 - 1.05) | 1.02 (0.49 - 2.09) |
| Primary | 1.00 (-) | 1.00 (-) | 1.00 (-) | 1.00 (-) |
| Secondary | 0.92 (0.67 - 1.26) | 0.90 (0.69 - 1.17) | ***1.21 (1.01 - 1.45)*** | 0.60 (0.26 - 1.39) |
| Tertiary | 0.77 (0.44 - 1.33) | 0.95 (0.48 - 1.86) | ***1.67 (1.12 - 2.48)*** | 0.92 (0.09 - 9.07) |
| Ethnic group |  |  |  |  |
| Malay | 1.00 (-) | 1.00 (-) | 1.00 (-) | - |
| Non-Malay | 0.98 (0.73 - 1.33) | 0.85 (0.63 - 1.14) | 0.88 (0.72 - 1.07) |  |
| Co-morbidities prior to stroke event | |  |  |  |
| Hypertension | 1.07 (0.85 - 1.36) | 1.16 (0.92 - 1.47) | ***2.39 (2.05 - 2.79)*** | ***-*** |
| Diabetes mellitus | 0.88 (0.71 - 1.08) | ***0.72 (0.58- 0.89)*** | 1.03 (0.91 - 1.18) | - |
| Dyslipidemia | ***1.40 (1.10 - 1.79)*** | ***1.28 (1.00 - 1.63)*** | ***0.84 (0.73 - 0.96)*** | ***-*** |
| Atrial fibrillation* | ***0.45 (0.32 - 0.63)*** | 1.07 (0.71 - 1.62) | 1.12 (0.86 - 1.46) | ***9.71 (2.05 - 46.05)*** |
| Ischemic heart disease | 0.84 (0.62- 1.14) | 1.12 (0.81 - 1.54) | 1.19 (0.99 - 1.43) | - |
| Previous stroke/TIA events | 0.81 (0.63 - 1.04) | 0.94 (0.74 - 1.20) | ***1.19 (1.02 -1.38)*** | ***-*** |
| Life-style factors |  |  |  |  |
| Smoking |  |  |  |  |
| Never smoked | 1.00 (-) | 1.00 (-) | 1.00 (-) | - |
| Previous smoker (quit >30 days) | 1.04 (0.71 - 1.52) | 0.96 (0.61 - 1.52) | 1.08 (0.87 - 1.34) |  |
| Current smoker | 0.98 (0.68 - 1.41) | 0.86 (0.61 - 1.23) | 0.99 (0.80 - 1.22) |  |
| Obesity | ***1.56 (1.00 - 2.42)*** | ***1.77 (1.15 - 2.73)*** | 1.04 (0.81 - 1.34) | - |
| Disability scale at discharge (mRS Score) |  |  |  |  |
| <3 | ***1.00 (-)*** | ***1.00 (-)*** | ***1.00 (-)*** | 1.00 (-) |
| >=3 | ***0.57 (0.45 - 0.71)*** | ***0.78 (0.63 - 0.97)*** | ***0.86 (0.75 - 0.98)*** | 0.61 (0.32 - 1.16) |
| Prior prescription of cardioprotective drugs |  |  |  |  |
| Antiplatelet drugs | ***2.43 (1.78 - 3.31)*** | ***0.74 (0.55 - 0.99)*** | 0.88 (0.73 - 1.06) | - |
| Anticoagulants | ***0.49 (0.28 - 0.85)*** | ***0.36 (0.19- 0.66)*** | 0.70 (0.44 - 1.14) | ***4.36 (2.23 - 8.51)*** |
| Antihypertensive drugs | 1.04 (0.82 -1.33) | 0.86 (0.68 - 1.09) | ***3.01 (2.59 - 3.49)*** | ***-*** |
| Lipid-lowering drugs | 1.00 (0.74 - 1.35) | ***3.25 (2.37 - 4.47)*** | 0.99 (0.82 - 1.19) | - |
| Types of hospitals† |  |  |  |  |
| State hospitals | 1.00 (-) | 1.00 (-) | 1.00 (-) | 1.00 (-) |
| Non-state hospitals | 2.27 (0.60 - 8.68) | 2.12 (0.55 - 8.19) | 1.07 (0.58 - 1.97) | 0.34 (0.09 - 1.30) |

*atrial fibrillation includes patients with documented history of atrial fibrillation and patients with electrocardiogram showing atrial fibrillation during admission for ischemic stroke

†state hospitals refer to hospitals with up to 45 resident specialties or subspecialties and are normally main referral centers for each state

Note: results were derived from multivariable analyses, ref. = reference groups, TIA= transient ischemic attack, mRS=Modified Rankin Scale, - = factors were not included in multivariable analysis
